# Dietary Chemoprevention of PhIP Induced Carcinogenesis in Male Fischer 344 Rats with Tomato and Broccoli

**DOI:** 10.1371/journal.pone.0079842

**Published:** 2013-11-27

**Authors:** Kirstie Canene-Adams, Karen S. Sfanos, Chung-Tiang Liang, Srinivasan Yegnasubramanian, William G. Nelson, Cory Brayton, Angelo M. De Marzo

**Affiliations:** 1 Department of Pathology, Johns Hopkins School of Medicine, Baltimore, Maryland, United States of America; 2 Department of Molecular and Comparative Pathobiology, Johns Hopkins School of Medicine, Baltimore, Maryland, United States of America; 3 Department of Urology, Johns Hopkins School of Medicine, Baltimore, Maryland, United States of America; 4 Department of Oncology, Johns Hopkins School of Medicine, Baltimore, Maryland, United States of America; University of Illinois, United States of America

## Abstract

The heterocyclic amine, 2-amino-1-methyl-6-phenylimidazo[4,5-B]pyridine (PhIP), found in meats cooked at high temperatures, has been implicated in epidemiological and rodent studies for causing breast, prostate, and colorectal cancers. A previous animal study using a xenograft model has shown that whole tomato and broccoli, when eaten in combination, exhibit a marked effect on tumor reduction compared to when eaten alone. Our aim was to determine if PhIP-induced carcinogenesis can be prevented by dietary consumption of whole tomato + broccoli powders. Male Fischer 344 rats (n = 45) were randomized into the following treatment groups: control (AIN93G diet), PhIP (200 ppm in AIN93G diet for the first 20 weeks of the study), or tomato + broccoli + PhIP (mixed in AIN93G diet at 10% each and fed with PhIP for 20 weeks, and then without PhIP for 32 weeks). Study animals were monitored for 52 weeks and were euthanized as necessary based on a set of criteria for health status and tumor burden. Although there appeared to be some hepatic and intestinal toxicity due to the combination of PhIP and tomato + broccoli, these rodents had improved survival and reduced incidence and/or severity of PhIP-induced neoplastic lesions compared to the PhIP-alone treated group. Rats eating tomato + broccoli exhibited a marked decrease in the number and size of cribiform prostatic intraepitheilial neoplasia/carcinoma *in situ* (cribiform PIN/CIS) lesions and in the incidence of invasive intestinal adenocarcinomas and skin carcinomas. Although the apparent toxic effects of combined PhIP and tomato + broccoli need additional study, the results of this study support the hypothesis that a diet rich in tomato and broccoli can reduce or prevent dietary carcinogen-induced cancers.

## Introduction

Prostate cancer (PCa) is the most commonly diagnosed non-cutaneous cancer in men and the second most deadly. The incidence of PCa in men in East and Southeast Asia is much lower than men in Western countries [1]. Since Asian men who immigrate to Western countries acquire an increased risk of PCa [2], environmental exposures likely contribute to prostate carcinogenesis in these men. Dietary practices may help to explain this difference since, as compared with an “Asian style” diet, a “Western style” diet is associated with an increased risk of cancer of the colon/rectum, breast and prostate [3]. One well-documented difference in the diets of men from these two cultures is that Westerners consume higher quantities of meat [4]–[7]. Heterocyclic amines are formed upon cooking meats at high temperatures, and a number of these compounds are potent carcinogens in rats. For example, exposure to 2-amino-1-methyl-6-phenylimidazo[4,5-b]pyridine (PhIP), the primary heterocyclic amine produced from cooking meat, results in cancer of the prostate, intestine, and mammary gland of rats [8]–[12]. Americans consume approximately 13.4 ng/kg/day of heterocyclic amines from well-cooked meats, with seventy percent being PhIP [13]. Epidemiological studies have indicated that men who consume high levels of PhIP have an increased risk of developing PCa, as well as an increased risk for metastatic disease and death [14]–[17]. Although PCa death rates have been dropping in both Caucasians and African-Americans, the rates for African-Americans are still more than double [18] that of Caucasians, possibly due in part to increased PhIP consumption in this demographic group [13], [17], [19], [20].

PhIP is widely believed to induce cancer after undergoing a number of metabolic activating events in which its metabolites adduct to DNA and cause DNA mutations. The prostate has been shown to activate N-hydroxy-PhIP, which occurs after N-hydroxylation in the liver, to genotoxic species, resulting in PhIP-DNA adducts [21]–[23]. However, the complete mechanism of carcinogenicity for PhIP is unknown. Nakai *et al*., [24] found that while all rat prostate lobes (ventral, dorsal, lateral and anterior) were target tissues for PhIP-related mutations, only the ventral prostate developed PIN (prostatic intraepithelial neoplasia) and early pre-invasive neoplastic lesions in response to PhIP exposure. Interestingly, only the ventral prostate showed increased proliferation and an influx of innate inflammatory cells consisting of mast cells and macrophages. This finding suggested that while the mutagenic effects of PhIP are necessary for PhIP-induced cancer formation, they are not sufficient. Thus, PhIP was acting as both an initiator and a lobe-specific cancer promoter [24].

Fruit and vegetable intake as a modifiable risk factor for cancer has been the subject of extensive epidemiologic investigation [25]–[28]. These studies have led to public health recommendations from the USDA [29] to make half your plate fruits and vegetables which “may be raw or cooked; fresh, frozen, canned, or dried/dehydrated; and may be whole, cut-up, or mashed” and the 2010 Dietary Guidelines [30] recommend to “eat a variety of fruits and vegetables, especially dark green and red and orange vegetables.” When similar nutritional guidelines are followed, there is a significant impact on the reduction of cancer incidence and mortality [31]. Findings from large prospective epidemiologic trials first raised awareness that tomatoes, particularly processed tomato products, consumed at a rate of approximately 5–7 servings per week were associated with a 30–40% reduction in prostate cancer risk [32]–[34]. In the Health Professionals Follow-Up Study (HPFS), an intake of ≥ two servings a week of tomato products compared to none resulted in a lower risk of prostate cancer, and a greater risk reduction for prostate cancer occurred with tomato sauce (RR  = 0.77 for 2+ servings/week versus < one serving/month; 95% CI  = 0.66 to 0.90; *P*_trend_ <0.001) than with lycopene intake alone (RR  = 0.84 for high versus low quintiles; 95% CI  = 0.73 to 0.96; *P*_trend_  = 0.003) [35]. A meta-analysis found that, compared to non-frequent consumers of raw tomatoes, the RR for prostate cancer in the highest quartile of intake was 0.89 (95% CI 0.80–1.00), and for those consuming cooked tomato products, the RR was 0.81 (95% CI 0.71–0.92) [36]. A relationship between cruciferous vegetables such as broccoli and prostate cancer emerged more recently from epidemiologic studies. The HPFS examined the effect of cruciferous vegetable consumption on prostate cancer risk and found a slight inverse association with organ-confined prostate cancer (RR, 0.88; 95% CI, 0.74–1.05, *P* for trend  = 0.06) [37]. In men under the age of 65, there was a stronger relationship between cruciferous vegetable intakes and decreased prostate cancer risk (RR, 0.81; 95% CI, 0.64–1.02, *P*_trend_  = 0.02), and for organ-confined prostate cancer (RR, 0.72; 95% CI, 0.54–0.97, *P*_trend_  = 0.007). The relationship was stronger when the analysis was restricted to men who consistently consumed cruciferous vegetables over the ten years prior to 1986 and had had a PSA test, indicating that cruciferous vegetables may be at least somewhat protective in regards to initiation of prostate cancer [37]. Kristal *et al*. [38] published a review paper examining the relationship of *Brassica* vegetable consumption and prostate cancer risk and found that three of the six well-designed studies reported significant reduced risks (p<0.05) and one reported a borderline significance for reduced risk (p = 0.06) with high *Brassica* vegetable consumption.

The antitumor effects of tomato and broccoli consumption on prostate cancer were previously tested in a rodent Dunning R3327H xenograft model of prostate cancer [39]. Interestingly, supplementing the rodents' diets with both 10% broccoli and 10% tomato powders produced a 52% decrease in Dunning R3327H prostate tumor weight compared to AIN93G control diet [39]. Since a rodent diet containing 10% tomato and 10% broccoli powders was shown to considerably reduce prostate tumor growth via increasing apoptosis and decreasing proliferation in this previous Dunning R3327H adenocarcinoma model [39], we hypothesized in the current study that tomatoes and broccoli could also reduce PhIP-initiated prostate tumors, thereby reinforcing the anticancer benefits of these functional foods.

## Materials and Methods

### Animals

**Figure 1:**
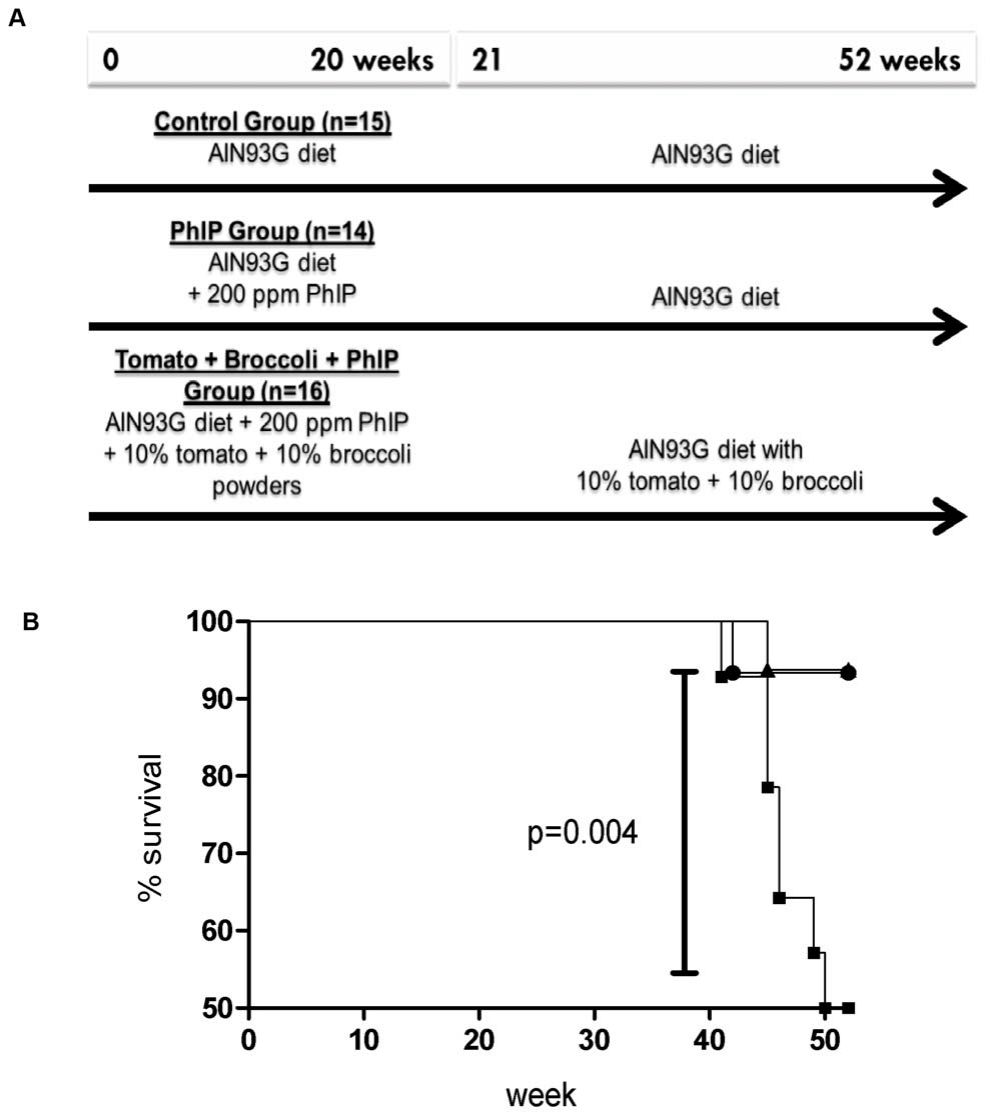
Study design and survival curve for 52 week study. A) Study design B) Survival curve. Control (•); PhIP (▪); tomato + broccoli + PhIP (▴).

All studies were approved by the Johns Hopkins Animal Care and Use Committee (Protocol Number: RA07M520). Male Fischer 344/NHsd rats (F344, Harlan, Frederick, MD, USA) aged 4 weeks were housed in pairs in polycarbonate cages with wire bottoms at the Johns Hopkins animal facilities and maintained on a 12 hour light-dark cycle, at a constant temperature (22±2°C) and relative humidity (55±15%). There were 3 groups in this study: control (n = 15), PhIP (n = 14), and tomato + broccoli + PhIP (n = 16) (Figure 1A). Tap water and rodent diets were available *ad libitum*, with animal body weights and feed intakes recorded weekly throughout the study. Factors to determine when euthanasia should occur were to diminish suffering and included loss of 10% body weight, hunched posture, bleeding which could not be controlled, lack of grooming, eating or drinking, or urine or fecal output, or a palpable tumor greater than 2 cm. Rats were euthanized by CO_2_ inhalation according to The American Veterinary Medical Association guidelines and the Johns Hopkins Animal Care and Use Committee guidelines on euthanasia.

### Diets

Diets were made by Bio-Serv, Inc. (Frenchtown, NJ, USA). All animals started on “control” AIN93G diet for one week before being randomized into treatment groups. The control group animals were maintained on the AIN93G diet for all 52 weeks of the study. The rest of the animals received the dietary carcinogen PhIP (PhIP hydrochloride obtained from the Nard Institute, Osaka, Japan), at a level of 0.2 g/kg (200 ppm) mixed into AIN93G purified rodent diet. Tomato and broccoli freeze dried powders were provided by Future*Ceuticals (Momence, Illinois, USA) and incorporated into AIN93G based diets at a level of 10% by weight for each powder. Diets were balanced for calories, fat, protein, carbohydrates, and fiber as described previously [39]. Tomato and broccoli powders were tested by Future*Ceuticals to be negative for coliforms, yeast, mold, *E. coli*, *Staph*, *Salmonella*, and *Listeria*. All diets were stored in air tight containers at 4°C in the dark. Fresh diet was provided to the rats on a weekly basis and food intakes were recorded. Animals received the PhIP or tomato + broccoli + PhIP containing diets for 20 weeks. The PhIP group was then moved onto AIN93G “control” diet without PhIP and the tomato + broccoli + PhIP group was moved onto tomato + broccoli diet for an additional 32 weeks. There was not a group of rats fed tomato and broccoli without PhIP as previous studies have shown no toxic effects of the diet [39].

### Serum Lipids

Serum lipids were measured using the Vet ACE® Clinical Chemistry System (Alfa Wasserman Diagnostic Technologies LLC; West Caldwell, NJ) at the Johns Hopkins Molecular and Comparative Pathobiology Phenotyping Core. In brief, the ACE® cholesterol assay measures both free cholesterol and cholesterol acyl esters; triglycerides were quantified by a modification of a fully enzymatic, single reagent procedure using glycerol phosphate oxidase and a modified Trinder reaction.

### Histopathology

Wet tissue weights were recorded for all collected tissues. All prostates were dissected separately into anterior, dorsal, lateral, and ventral lobes. In addition, the seminal vesicles, bladder, liver, kidney, spleen, and any macroscopically abnormal tissues were immediately dissected and placed into formalin. All of these tissues were fixed in 10% buffered formalin for 48 hours before paraffin embedding. The small intestines and colon were flushed with 1× PBS and formalin to remove contents before fixation in 10% buffered formalin for 48 hours and paraffin embedding. Hematoxylin and Eosin (H&E) staining was performed using the Leica Microsystems AutoStainer XL (Buffalo Grove, IL). Slides were scanned at 20× using the Aperio ScanScope (CS model, Aperio, Vista, CA) and viewed using the freeware ImageScope Viewer Software (Aperio version 10.2.2.2353). The entirety of the ventral prostate area was evaluated on two separate histological tissue sections per animal along with the area of cribiform PIN/carcinoma *in situ* (PIN/CIS) to calculate the mean percentage area of cribiform PIN/CIS lesions in the PhIP-alone and tomato + broccoli + PhIP group at 52 weeks. These two tissue sections were selected fifty microns apart.

### GTSP1 Immunohistochemistry

Immunohistochemistry was performed using the Power Vision+ Kit (Leica Microsystems, Buffalo Grove, IL). Briefly, GSTP1 primary antibody incubation (Assay Design, Plymouth Meeting, PA) at a dilution of 1∶10,000 in Antibody Dilution Buffer, (Ventana, Tuscan, AZ) was carried out overnight at 4°C. Powervision Poly-HRP anti-rabbit IgG secondary antibody incubation was carried out for 30 minutes at room temperature. Histochemical localization was carried out using 3,3′-diaminobenzidine tetrahydrochloride (DAB, Sigma) and counterstained with hematoxylin.

### Statistics

Unless otherwise noted, data were compared among treatments by two-tailed Mann-Whitney U tests for non-parametric data using GraphPad Prism Software (version 5.0; GraphPad Software, Inc., La Jolla, CA), and values were considered significantly different at p<0.05. Testes weights were analyzed via ANCOVA using IBM SPSS Statistics 21(Armonk, New York).

## Results

### Body Weights and Food Intakes

**Figure 2:**
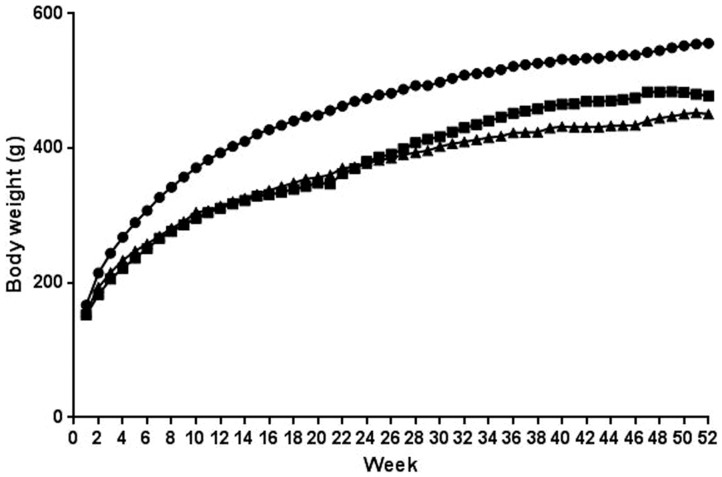
Average body weight of study animals over 52 weeks. Control (•); PhIP (▪); tomato + broccoli + PhIP (▴).

Animals were randomized into groups with approximately equal body weight averages: control 131.5±2.1 g (± standard error), PhIP 134.1±2.5 g, and tomato + broccoli + PhIP 135.7±2.9 g. After two weeks of consumption of PhIP-containing diets, the body weights in the PhIP-alone and tomato + broccoli + PhIP groups significantly diverged from control rats (p<0.0004), but did not differ significantly from each other (p = 0.052, Figure 2). The trend for PhIP fed groups having the smallest body weights was maintained until the completion of the study at 52 weeks (478.1±14.5 g, p = 0.0003 for PhIP-alone treated rats and 451.0±8.1 g, p<0.0001 for the tomato + broccoli + PhIP treated rats) compared to control (556.2±8.3 g), but did not differ significantly from each other (p = 0.066).

The PhIP model of carcinogenesis is a well-established model for studying the effect of PhIP consumption on prostate and gastrointestinal tumors in male rats. Prostate studies use a range of 25 to 400 ppm PhIP either incorporated into the diet or intragastrically for ten to 104 weeks [40]–[45]. Borowsky *et al*. [41] reported decreased food intakes, body weights, and health of rats consuming 400 ppm PhIP for 20 weeks, thus we chose to use 200 ppm for 20 weeks in our study. Yet, even at this decreased PhIP dose, a decrease in both body weights and food intakes were seen in all PhIP consuming rats in our study. From weeks 0 through 20, the PhIP-alone group rats consumed an average of 14.9±0.2 g/day (± standard error), the tomato + broccoli + PhIP group ate 15.0±0.2 g/day, and the control group ate 17.0±0.2 g/day, with significant differences between PhIP-containing diet groups versus the control group, p<0.0001. As per our study design, from weeks 21–52, the PhIP group was put on control diet and PhIP was removed from the tomato + broccoli diet (Figure 1A). During this time, the PhIP-alone, tomato + broccoli + PhIP, and control groups consumed 15.5±0.2, 15.1±0.1, 16.8±0.1 g/day, respectively, with significant differences between PhIP-containing diet groups versus the control group, p<0.0001. There were no significant differences at any time point between the PhIP-alone and tomato + broccoli + PhIP group food intakes. Since there were no significant differences in body weight or food intake between the PhIP-alone group and the tomato + broccoli + PhIP group, the decrease in food intake and body weight in these groups was unlikely the cause of any observed differences in tumor size or incidence between the two groups (see below).

### Reproductive Tissue Weights

Teste weights were evaluated based on analysis of covariance as suggested by Bailey *et al*. and no significant differences were seen among groups, Table 1 [46]. Ventral, anterior, and dorsolateral prostate lobes of PhIP-alone treated animals were significantly lower than control or tomato + broccoli + PhIP animals (Table 1, p<0.02). Seminal vesicle tissue weights of PhIP-alone treated animals were significantly lower than control group animals.

**Table 1:** Mean Tissue Weights of Fischer 344 Male Rats ± Standard Error of the Mean.

Treatment Group	VP	AP	DLP	SV	Testes
	% body weight				Grams
Control	0.08±0.01	0.06±0.01	0.10±0.01	0.25±0.01	3.18±0.2
PhIP	0.05±0.01*	0.04±0.003*	0.07±0.01*	0.18±0.02*	3.0±0.3
T+B+ PhIP	0.07±0.003*,**	0.06±0.01**	0.10±0.01**	0.22±0.02	3.1±0.3

*statistically different from control group p<0.02;

**statistically different from PhIP group p<0.02.

VP  =  ventral prostate, AP  =  anterior prostate, DLP  =  dorsolateral prostate, SV  =  seminal vesicle.

### Animal Survival

As shown in Figure 1B, the animals consuming PhIP alone exhibited decreased survival beginning at 41 weeks. In contrast, by the end of 52 weeks, only one animal in the control group and one animal in the tomato + broccoli + PhIP group died or had to be euthanized. The control animal was found dead of unknown causes and the tomato + broccoli + PhIP-treated animal had to be euthanized due to a histiocytic sarcoma. At the end of 52 weeks, 14 of 15 (93.3%) control animals, 15 of 16 (93.8%) tomato + broccoli + PhIP animals and 7 of 14 (50%) PhIP-alone treated animals survived (Figure 1B). The survival curves of the control and tomato + broccoli + PhIP groups were significantly improved compared to the PhIP-alone group (p = 0.004, Chi square 11.09, long-rank Mantel-Cox comparison).

### Ventral Prostate Tumor Incidences

**Figure 3:**
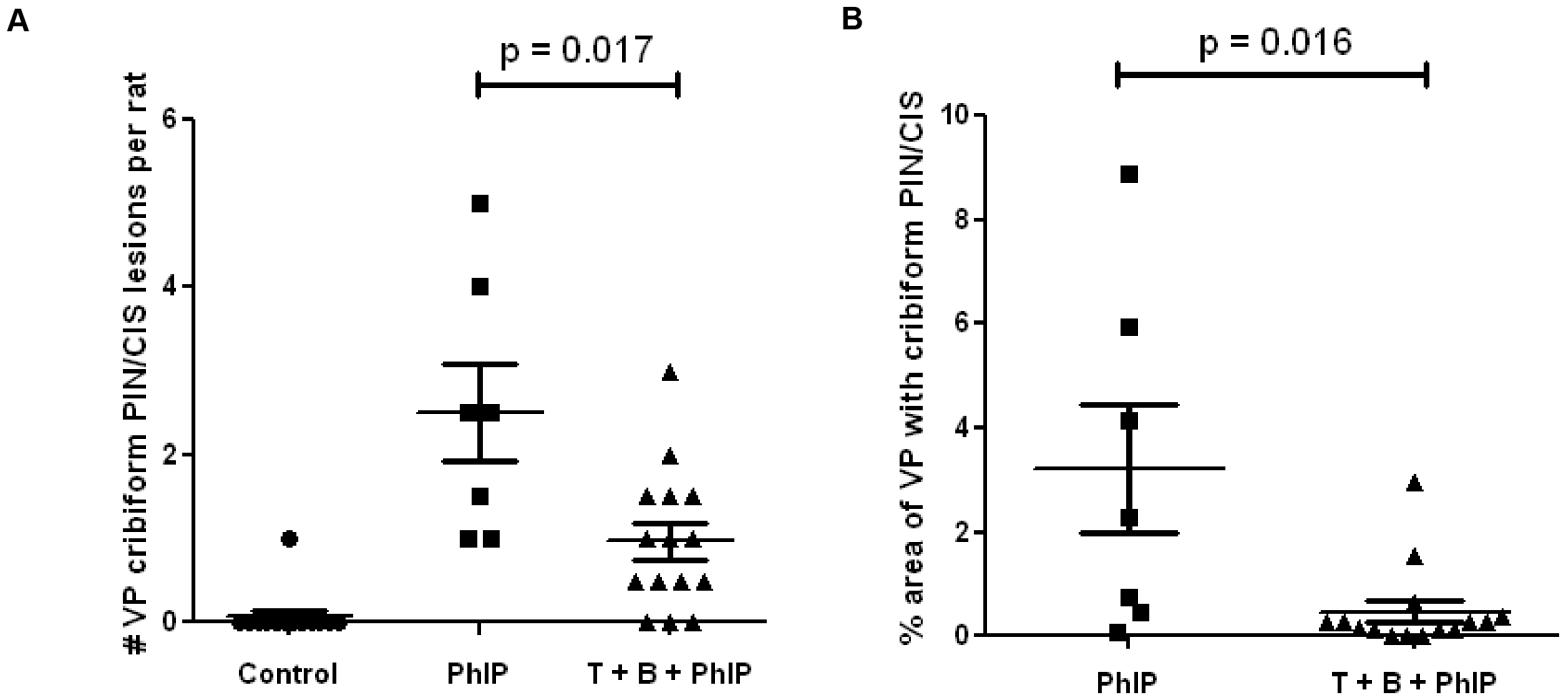
Tomato + broccoli in combination with PhIP results in a significant decrease in number and size of ventral prostate (VP) cribiform PIN/CIS lesions. A) Number of VP cribiform PIN/CIS lesions (end of study animals at 52 weeks). B) Size of cribiform PIN/CIS lesions as percent area of VP (end of study animals at 52 weeks). Two H&E step sections were analyzed per animal and average values are reported.

**Figure 4:**
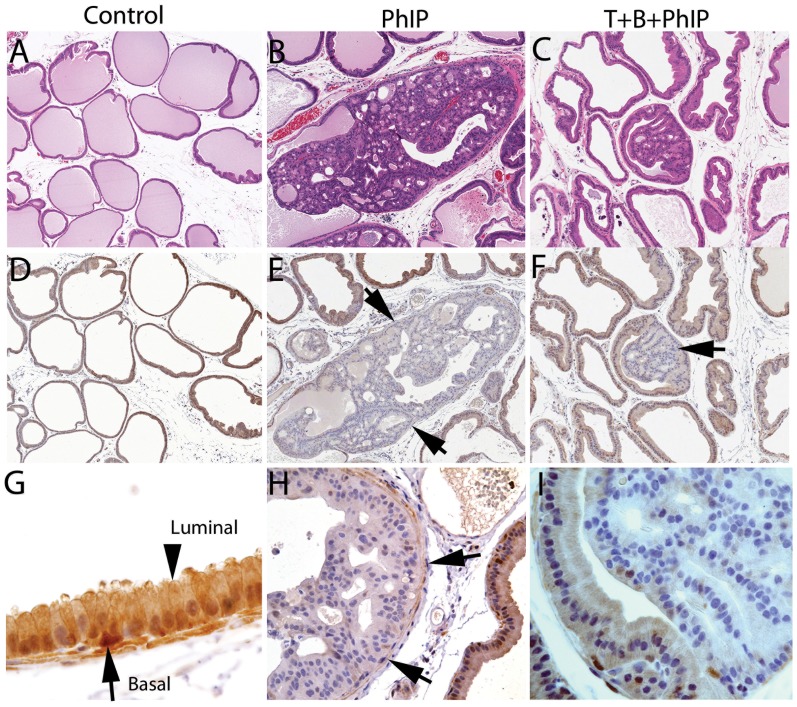
Loss of GSTP1 in ventral prostate cribiform PIN/CIS lesions. Representative H and E images of ventral prostates from A) Control B) PhIP and C) tomato + broccoli + PhIP rats. D–F, IHC for GSTP1. Note loss of GSTP1 staining in cribiform PIN/CIS lesions in PhIP-alone (E) and tomato + broccoli + PhIP-treated (F) rats. G, Higher power view (400× original magnification) of region of normal epithelium from D showing robust staining for GSTP1 in luminal cells (arrowhead) and even stronger staining in basal cells (arrow). Arrows in E, F, and H denote a very smooth rounded border for the cribiform PIN/CIS glands and an intact smooth muscle layer, indicating that there is no stromal invasion.

One spontaneous ventral prostate cribiform PIN/CIS lesion was observed in an animal in the control diet group. In comparison, consumption of PhIP-alone at 200 ppm for 20 weeks resulted in 92.9% of the rats developing ventral prostate cribiform PIN/CIS lesions. The consumption of tomato + broccoli in conjunction with PhIP diet resulted in an incidence of cribiform PIN/CIS lesions of 81.3%, which was not significantly different from the PhIP alone group (p = 0.60 by Fisher's Exact test). It should be noted that the average time to death for PhIP-alone treated animals was 49±3.7 weeks, whereas only one tomato + broccoli + PhIP animal had to be euthanized before the end of the 52 week study due to tumor burden (Figure 1B). Therefore, cribiform PIN/CIS lesions in tomato + broccoli + PhIP animals had a longer period of time to develop on average. When the extent of cribiform PIN/CIS was compared in only those animals that survived to the end of study (52 weeks), the number of lesions per ventral prostate was reduced to 1.0 per rat fed tomato + broccoli + PhIP compared to rats fed PhIP-alone which had 2.5 lesions per ventral prostate, p = 0.017 (Figure 3A). Area analysis was performed to quantify the percentage of the ventral prostate that was comprised of cribiform PIN/CIS lesions. The results of this analysis revealed a>6-fold reduction in the extent of neoplastic lesions in tomato + broccoli + PhIP animals compared to PhIP treated animals (0.5% of ventral prostate comprised of cribiform PIN/CIS versus 3.2%, respectively), p = 0.016 (Figure 3B, Figure 4A–C).

### GSTP1 Immunohistochemistry

Glutathione S-transferase pi 1 (*GSTP1*) is known to be silenced by hypermethylation of deoxycytidine residues within CpG dinucleotides within its upstream regulatory region in nearly all human prostate cancers [47], [48]. GSTP1 protein is expressed most highly in the basal cells of the normal human prostate, with generally little or no expression in normal luminal secretory epithelial cells. GSTP1 protein is, however, expressed at high levels at times in human prostatic luminal epithelial cells, apparently in response to cellular stress and/or inflammatory oxidants in atrophic lesions referred to as proliferative inflammatory atrophy (PIA) [49], and we have previously shown that these atrophic luminally located cells are not basal cells as they lack p63 expression [50]. Additional evidence that shows that these apparently stressed luminal cells are not simply basal cells is also provided by the fact that they frequently express GSTA1, which is generally not expressed in normal basal cells [51].In mice, we have previously reported that GSTP1 shows a different pattern of expression in which it is not expressed at high levels in basal cells and low levels in luminal cells; rather it is expressed in both compartments at relatively equal levels [52]. Interestingly, when *Gstp1/2* homozygous knockout mice were “humanized” at the *GSTP1* locus by generating transgenic animals containing the human gene along with its upstream regulatory region, the expression pattern in these mice mimicked the human in which there is much higher levels of staining in the basal compartment and little or no staining in the luminal cells in normal regions [52]. Rats appear somewhat different than humans and mice in their expression of GSTP1 in the ventral prostate since like mice, they show relatively moderate levels of expression in most luminal cells, but like humans, they show stronger expression in basal cells (Figure 4G). Figure 4H–I shows that while benign normal appearing epithelium (basal and luminal cells, although mostly luminal cells are visible) expresses GSTP1, the cribriform PIN/CIS lesions showed marked reductions in staining. This reduced GSTP1 staining in non-invasive cribriform neoplastic lesions was also reported by Borowsky et al. [41] and in a recently described CYP1A-humanized mouse model [53].

### Skin Tumor Incidence

**Figure 5:**
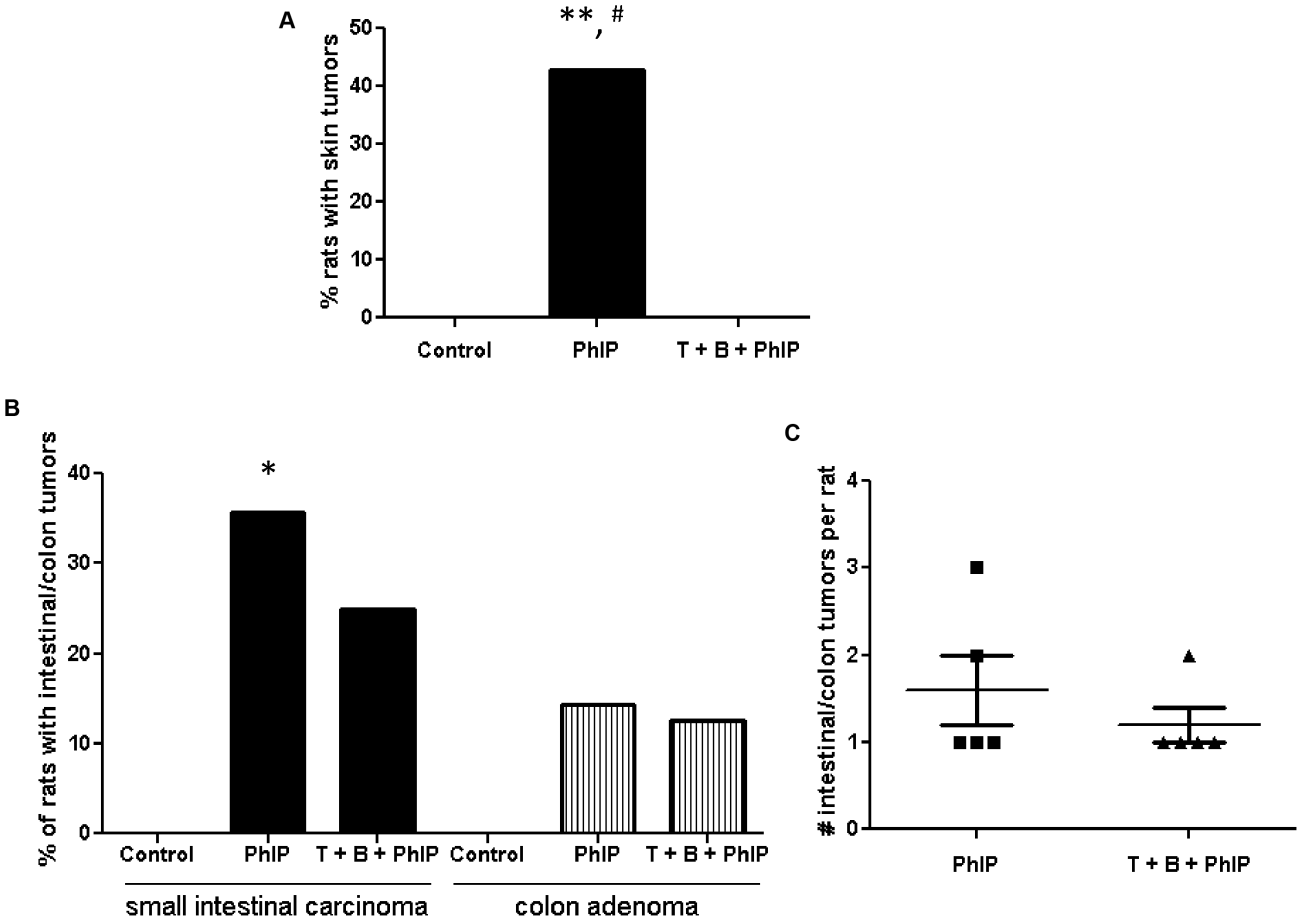
Tomato + broccoli in combination with PhIP results in a decrease in skin and gastrointestinal tumor incidence. A) Incidence of skin tumors (all study animals). B) Incidence of small intestinal carcinoma and colon adenoma (all study animals). C) Number of gastrointestinal tumors per rat (of study animals with gastrointestinal tumors). * Significant difference between control and PhIP group (Fisher's exact test, p = 0.02), ** Significant difference between control and PhIP group (Fisher's exact test, p = 0.006), ^#^Significant difference between PhIP and tomato + broccoli + PhIP group (Fisher's exact test, p = 0.005).

PhIP-treated male Fischer 344 rats are known to develop tumors at sites in addition to the prostate including the skin, small intestines and colon [54]. In regards to skin tumors, 42.9% of PhIP-alone treated animals developed lesions. These lesions were a mixture of invasive and pre-invasive basaloid, sebaceous, and squamous tumors. In contrast, control animals and, strikingly, tomato + broccoli + PhIP animals did not develop any skin tumors (Figure 5A).

### Intestinal and Colon Tumor Incidence

AIN93G control diet-fed rats had no tumors in the gastrointestinal tract. In the PhIP-alone and tomato + broccoli + PhIP groups there was an incidence rate of 35.7% and 25%, respectively, of invasive small intestinal carcinoma (Figure 5B) compared to animals on the control diet. Adenomas were also observed in the colon of PhIP-alone (14.3%) and tomato + broccoli + PhIP-treated animals (12.5%). The number of gastrointestinal tract neoplastic lesions seen per rat decreased from 1.6 to 1.2 lesions per rat, although this was not statistically significant (Figure 5C). It should be noted that many of the PhIP-alone treated animals with gastrointestinal tumors did not survive to the end of the 52 week study (average time to death of 46.8±4.3 weeks). In all but one of the PhIP-alone treated animals with gastrointestinal tumors, the animal either died or had to be euthanized early due to this tumor. In stark contrast, none of the tomato + broccoli + PhIP animals died or needed to be euthanized early due to gastrointestinal tumors.

### Enlarged Spleens and Livers in Tomato + Broccoli-Fed Rats

**Figure 6:**
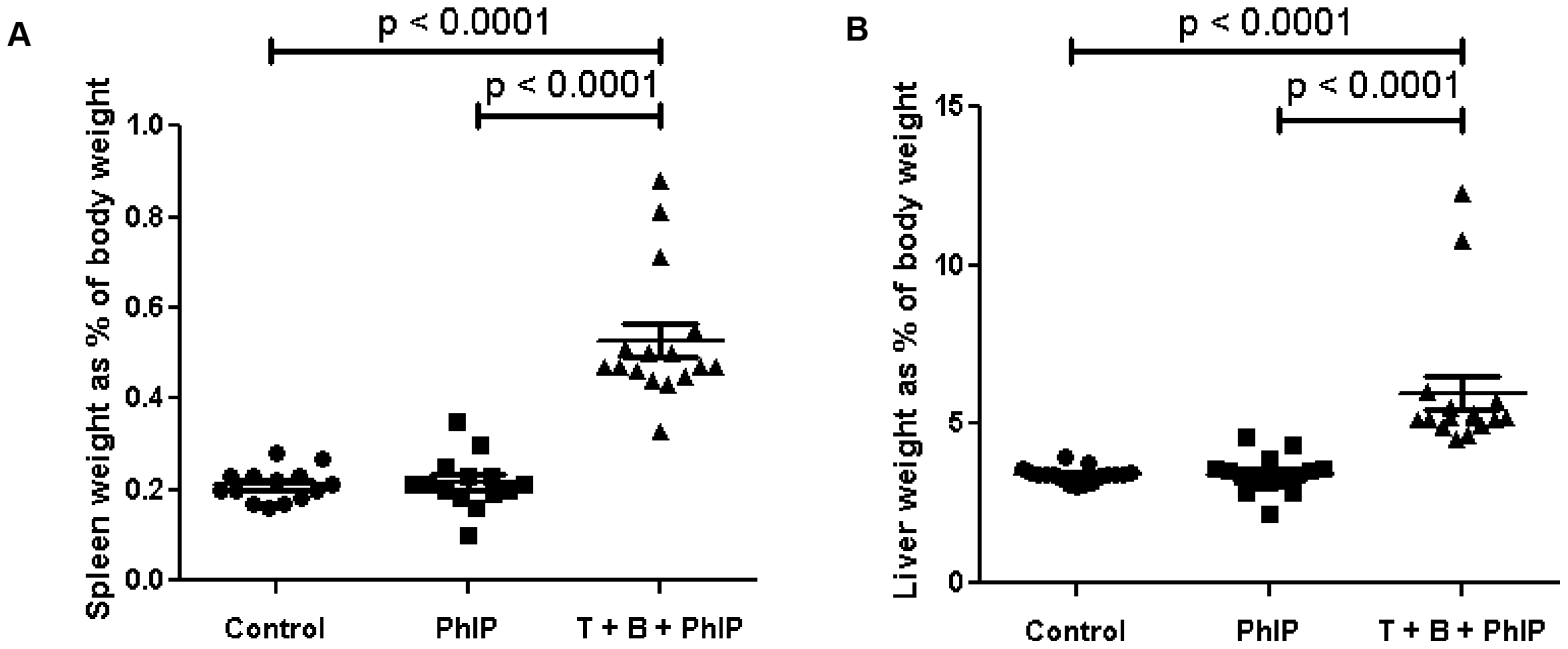
Increase in spleen and liver weight in tomato + broccoli fed rats. A) Spleen weight as percent of rat body weight. B) Liver weight as percent of rat body weight.

**Figure 7:**
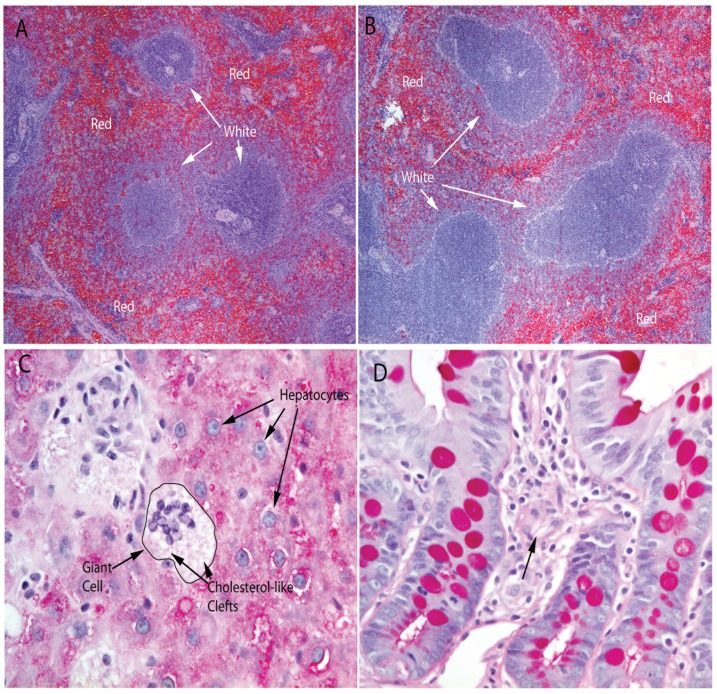
A–B) Representative H&E images of spleens from control rats (A), tomato + broccoli + PhIP rats (B). Note expansion of white pulp in tomato + broccoli + PhIP-treated rat. C) PAS stain showing example of infiltration of giant cells into liver of tomato + broccoli + PhIP-treated rats seen on H & E. D) Small intestine of a tomato + broccoli + PhIP-treated rat seen with PAS stain with cholesterol-like clefts indicated (arrow).

An unexpected finding in rats fed a diet enriched for tomato + broccoli + PhIP was significantly enlarged spleens and livers compared to control animals and PhIP-alone treated animals (Figure 6A,B). Tomato + broccoli + PhIP spleens and livers were larger than the control and PhIP tissues, p<0.0001. Spleens were observed to have expansions of white pulp (Figure 7A–B) and, intriguingly, livers were observed to often contain mononuclear cells, or multinucleated cells resembling giant cells, with abundant cytoplasmic accumulations of structures similar to cholesterol clefts (Figure 7C). These cholesterol cleft-like structures were also observed in the small intestines of tomato + broccoli + PhIP-fed rats (Figure 7D), but were present in very limited areas of the spleens. We tested serum lipid levels in the study animals and the tomato + broccoli + PhIP group had significantly lower serum triglycerides (149.2±10.4) than the control (420.9±47.4) and PhIP-alone groups (463.2±67.0), p<0.0001 (Table 2). Serum cholesterol and HDL levels in the tomato + broccoli + PhIP group were significantly higher than that of the PhIP group, p = 0.03 and p = 0.01, respectively; however, there were no significant differences between serum cholesterol and HDL between the tomato + broccoli + PhIP group and the control group (Table 2). There were no significant differences between serum triglycerides, cholesterol, or HDL between the control and PhIP groups (Table 2).

**Table 2:** Mean Serum Lipid Levels of Fischer 344 Male Rats at end of 52 Week Study.

Treatment Group	Triglycerides (mg/dl ± SEM)	Cholesterol (mg/dl ± SEM)	HDL (mg/dl ± SEM)
Control	420.9±47.4	196.3±10.6	34.1±1.2
PhIP	463.2±67.0	158.0±11.9	30.7±1.0
T+B+ PhIP	149.2±10.4*	201.6±8.9**	36.7±1.4***

*p<0.0001 from control and PhIP serum levels;

**p = 0.03 from the PhIP group serum levels.

***p = 0.01 from the PhIP group serum levels.

Lipid levels were measured in control rats (n = 8), PhIP-treated rats (n = 6) and tomato + broccoli + PhIP (T+B+ PhIP)-treated rats (n = 9) chosen at random.

## Discussion

Cancer biologists have typically approached nutrition and cancer risk with a “reductionist” approach. The majority of cell biology and experimental carcinogenesis studies have examined a single specific chemical component derived from fruits and vegetables. The alternative approach, focusing upon whole foods, has been less vigorously pursued, due to the chemical complexity of specific foods, variability in composition, difficulty in characterizing mechanism(s) of action, and perceived obstacles to completing randomized clinical trials with whole foods. While the more reductionist approach is scientifically appealing, in general single-agent trials have proven to be disappointing, implying that in isolation, many compounds do not appear to behave as expected in laboratory studies. For example, beta-carotene studies led to negative results in two phase III randomized cancer prevention trials, the Alpha-Tocopherol, Beta-Carotene Cancer Prevention (ATBC) Study [55] and the Carotene and Retinol Efficacy Trial, CARET [56]. In the current study, we chose to feed rats whole tomato and broccoli powders at levels a human could easily consume and thus physiologically relevant. Based on body weight and basal metabolic rates of rats versus humans, we have calculated that a human male would need to eat less than a half cup of tomato paste, one cup of tomato sauce, or two and a half cups raw tomatoes and less than a cup and a half of broccoli to equal the amounts the rats received in this study. Tomato products are good sources of potassium, folate, and the vitamins A, C, and E, the carotenoids lycopene, β-carotene, phytoene, and phytofluene, the flavonols quercetin and kaempferol, and polyphenols. Broccoli contains folate, potassium, and the vitamins A, C, E, and K, the carotenoids α-, β–carotene, lutein, and zeaxanthin, and the polyphenol quercetin. Broccoli also contains a class of compounds called glucosinolates, which undergo hydrolysis to form three major classes of products: isothiocyanates, nitriles, and thiocyanates. Glucoraphanin is converted to sulforaphane via the enzyme myrosinase when broccoli is crushed or chewed. Glucobrassicin forms indole-3-carbinol (I3C) which, in the acidic environment of the stomach, can be converted to a number of acid condensates including 3,3″-Diindolylmethane (DIM).

Previous work has demonstrated the antitumor effects of tomato + broccoli in the Dunning R3327-H prostate adenocarcinoma model [39]. In the present study, the frequency and size of PhIP-induced ventral prostate cribiform PIN/CIS lesions in Fischer 344 rats were significantly reduced with concurrent intake of 10% tomato +10% broccoli in the diet. These results demonstrate that a diet rich in tomato + broccoli can have a significant impact on dietary carcinogen-induced prostate carcinogenesis. As in previous studies of PhIP-induced prostatic neoplasia in rodents [41], [53], we found that the GSTP1 protein, which is silenced via promoter methylation in ∼90% of human prostate cancers, was decreased in cribiform PIN/CIS lesions in the rat ventral prostate. We do not currently know if the absence of GSTP1 in PhIP-induced rat ventral prostate cribiform PIN/CIS lesions is related to DNA hypermethylation of the *GSTP1* promoter region as it is in the human, and this represents an area for future studies. However, even without knowing the precise mechanism of inactivation, the findings of clearly reduced GSTP1 protein levels in the all of the prostatic neoplastic lesions adds to the overall relevance of this model system.

In addition to the effects that were observed with consumption of tomato + broccoli on PhIP-induced prostate carcinogenesis in this study, we also observed a profound effect on other known sites of PhIP-induced cancers. For example, not a single skin cancer developed in the rats fed tomato + broccoli + PhIP (in comparison to 43% of the PhIP-alone treated animals). There was also a non-significant decrease in the incidence of invasive small intestinal cancer in rats fed tomato + broccoli in combination with PhIP. There was also an apparent reduction in the severity of these tumors, as indicated by the fact that most of the PhIP-alone treated animals with small intestinal tumors died or had to be euthanized before end of study, whereas no tomato + broccoli + PhIP-treated animals died early due to small intestinal tumors. The results of these studies are consistent with a more generalized anticancer mechanism of these whole foods on PhIP-induced carcinogenesis.

An unexpected finding in animals consuming PhIP + tomato + broccoli-enriched diets was apparent toxicity which was manifested as enlarged livers and spleens and the presence of cholesterol cleft-like structures in livers and small intestines (Figure 7). Tomato and broccoli powders obtained from Future*Ceuticals (Momence, Illinois, USA) underwent routine quality assurance testing for standard plate count (SPC) and the presence of coliforms, yeast and mold, *E. coli*, *Staphylococcus, Salmonella,* and *Listeria*. All tests were either negative or well below threshold levels, indicating that microbial contamination of the tomato and broccoli powders likely could not explain the liver and spleen phenotypes. The presence of cholesterol cleft-like structures could also not be explained by elevated serum lipid levels in tomato + broccoli-fed rats (Table 2). Serum triglycerides were actually significantly lower in the tomato + broccoli + PhIP-treated rats than in control or PhIP-alone treated rats. Lowered serum triglycerides in broccoli-fed rats has in fact been previously reported [57]. Likewise, HDL and total cholesterol was similar in tomato + broccoli + PhIP-treated rats to that of control animals. This liver and spleen phenotype (nor any other apparent toxicity) was not observed in previous studies in male Copenhagen rats fed a diet of 10% tomato +10% broccoli [39]. Given this lack of toxicity in this prior study, unfortunately we did not include a control group fed tomato + broccoli alone in the present study. Thus, the effect may either be strain specific to Fischer 344 rats, or may result from PhIP treatment in combination with tomato + broccoli. It should be noted that the dose of 200 ppm PhIP utilized in this study has been selected for a high incidence of rodent cancers and is not a physiologically relevant dose compared to human PhIP consumption levels. Thus, if the combination of tomato + broccoli + PhIP is resulting in generation of a toxic compound or compounds, this would not be necessarily expected to occur in humans due to the lower levels of PhIP consumed by humans routinely. Nevertheless, additional studies need to be carried out to determine if tomato + broccoli alone or the combination of PhIP treatment with tomato + broccoli is responsible for the observed phenotype in Fischer 344 rats.

## Conclusions

The results from this study demonstrate that intake of whole foods, such as broccoli and tomato, may significantly reduce dietary carcinogen-induced prostate and skin cancers. The rodent diet in this study contained 10% tomato and 10% broccoli powders which is equivalent to a 1 cup serving size of tomato sauce and 1.4 cups of broccoli for a human. Therefore, while there was some apparent toxicity with the combination of PhIP with tomato and broccoli that requires additional study, our findings support the public health approach to diet and cancer prevention exemplified by the New American Plate, the Dietary Guidelines for Americans, and Myplate.gov supported by the AICR, CDC, USDA, and NIH/NCI that recommend the consumption of a variety of colored fruits and vegetables on half of our breakfast, lunch, and dinner plates in order to reduce cancer risk [29], [30], [58].
