# Correction: Dietary Chemoprevention of PhIP Induced Carcinogenesis in Male Fischer 344 Rats with Tomato and Broccoli

**DOI:** 10.1371/annotation/1ac6412e-619f-4575-8b63-f5e16bd3252b

**Published:** 2014-01-07

**Authors:** Kirstie Canene-Adams, Karen S. Sfanos, Chung-Tiang Liang, Srinivasan Yegnasubramanian, William G. Nelson, Cory Brayton, Angelo M. De Marzo

The affiliation for the third author is incorrect. Chung-Tiang Liang is not affiliated with institution number 2, but rather the following institution: National Laboratory Animal Center, National Applied Research Laboratories, Taiwan.

