# Correction: Dietary Chemoprevention of PhIP Induced Carcinogenesis in Male Fischer 344 Rats with Tomato and Broccoli

**DOI:** 10.1371/annotation/3fd9703c-a6d9-4715-9c93-cf5279ea4fa6

**Published:** 2014-01-07

**Authors:** Kirstie Canene-Adams, Karen S. Sfanos, Chung-Tiang Liang, Srinivasan Yegnasubramanian, William G. Nelson, Cory Brayton, Angelo M. De Marzo

There is an error in the Competing Interests statement. The Competing Interests statement should read: 

"AMD is currently a Professor of Pathology, Urology and Oncology at the Johns Hopkins University School of Medicine (Baltimore, Maryland) and was also employed as such during the conduct of this study. During part of the writing of this manuscript AMD was employed at Predictive Biosciences, Inc. (Lexington, Massachusetts), as well as part-time adjunct Professor of Pathology, Oncology, and Urology at the Johns Hopkins University School of Medicine (Baltimore, Maryland). No funding or other support was provided by Predictive Biosciences, Inc., for any of the work in this manuscript. The terms of the relationship between AMD and Predictive Biosciences, Inc., were managed by the Johns Hopkins University in accordance with its conflict-of-interest policies. This does not alter the authors’ adherence to all the PLOS ONE policies on sharing data and materials."

